# Aneuploidy and Drug Resistance in Pathogenic Fungi

**DOI:** 10.1371/journal.ppat.1003022

**Published:** 2012-11-15

**Authors:** Kyung J. Kwon-Chung, Yun C. Chang

**Affiliations:** Molecular Microbiology Section, Laboratory of Clinical Infectious Diseases, National Institute of Allergy and Infectious Diseases, National Institutes of Health, Bethesda, Maryland, United States of America; Duke University Medical Center, United States of America

Genomic imbalance resulting from aneuploidy is detrimental to the proliferation of cells in conditions suitable for normal growth and differentiation [1], [2]. However, there is growing evidence that aneuploidy confers a significant growth advantage when cells undergo severe genetic or environmental perturbations [3]–[6]. For example, aneuploidy restored the ability to proliferate in yeast cells that carried an irreversible disruption in the key cytokinetic machinery [7], enabled them to overcome nutrient limitations [8], facilitated resistance to antifungal drugs in pathogenic fungi, and increased their virulence [4], [9], [10]–[12]. This review focuses on the emergence of aneuploidy that enables pathogenic fungi to resist antifungal drugs.

## What Is Aneuploidy and How Is It Produced?

Cells possessing a chromosome number that is either more or less than the normal number (wild type) are called aneuploids, which occur either spontaneously or as a consequence of genetic or environmental perturbation [1]. Although the cell division cycle is a highly controlled process with checkpoints to ensure the formation of two genetically identical daughter cells, chromosome missegregation does occur spontaneously at certain rates and produces aneuploids. In *Saccharomyces cerevisiae*, aneuploidy occurs spontaneously once every 5×10^5^ cell divisions [13], but this rate can increase more than a 100-fold when the cells are under certain environmental stress conditions [10]. While the spontaneous rate of aneuploidy in mammalian cells is in the order of 10^4^–10^5^ cell divisions [14], the majority of human solid tumor cells are aneuploids [15]. Whether aneuploidy occurs spontaneously or due to genetic or environmental factors, chromosome missegregation during cell division can occur by defects present in any one of the following:

1. structural integrity of the microtubule spindle apparatus
2. organization of spindle microtubules in bipolar arrays with focused poles
3. integrity of kinetochores on chromosomes
4. binding of spindle microtubules on kinetochores
5. checkpoint signaling pathway
6. process of cytokinesis (reviewed by [16])

Aneuploidy is poorly tolerated unless it provides a fitness advantage under stress [2], [4], [5] or carries aneuploidy-tolerating mutations. For example, the budding yeast cells with mutations in their deubiquitinating enzyme tolerate aneuploidy by attenuating the changes in intracellular protein composition resulted from aneuploidy [17].

## What Is Known about Aneuploidy and Resistance to Antifungal Drugs in Pathogenic Fungi?

Fungal infections are treated primarily with antimycotic agents that affect membrane sterols (e.g. polyenes) or disrupt biosynthesis of nucleic acids (e.g. 5-fluorocytosine), ergosterol (e.g. azoles), or cell wall components (e.g. echinocandins) [18], [19]. Prolonged use of these agents has been associated with an increase in the number of clinical cases exhibiting resistance to these drugs, which often correlates with in vitro resistance. Reports associating aneuploidy with the emergence of antifungal drug resistance in pathogenic fungi, however, have mostly been limited to azoles, primarily fluconazole (FLC), though polyenes and echinocandins have just as commonly been used as azoles. FLC is a fungistatic drug most widely used for yeast infections such as candidiasis and cryptococcosis. Consequently, reports of aneuploidy in pathogenic fungi linked to the emergence of drug resistance have been limited to FLC therapy, and studies of its mechanism have exclusively been carried out in *Candida* and *Cryptococcus*. The prevalence of azole-associated aneuploidy in these fungi appears not only to be due to increased azole therapy but also the high plasticity of their genomes [5], [20].

## How Does Aneuploidy Provide Fitness to *Candida* Species under Azole Stress?

The association between acquired aneuploidy and FLC resistance in *Candida* was first reported in a strain of *C. glabrata* isolated from a patient after just nine days of treatment for candidiasis with 400 mg FLC [21]. The resistant strain contained twice the levels of microsomal cytochrome P450 and ergosterol than the pretreatment strain. Repeated subcultures of the resistant strain on drug-free media decreased both the P450 content and ergosterol synthesis while restoring FLC susceptibility. Analysis of chromosome patterns revealed that the whole chromosome harboring *ERG11* (*CYP51*), which encodes the azole target, had been duplicated in the resistant strain. The extra copy of the duplicated chromosome along with FLC resistance gradually disappeared during growth on drug-free media, suggesting an association between duplication of the *ERG11*-bearing chromosome and drug resistance [22]. Although formation and loss of the new chromosome associated with FLC resistance was observed in more clinical isolates, details on the FLC-induced aneuploidy in *C. glabrata* have not been pursued [11]. Acquisition of aneuploidy-conferring azole resistance, however, was extensively studied in *C. albicans* by Berman and associates [4], [20], [23], [24]. Analysis of a large number of azole-resistant and azole-sensitive strains from clinical and laboratory sources by comparative genome hybridization (CGH) revealed a clear link between aneuploidy and azole resistance [23]. Aneuploidy was most prevalent for chromosome 5 (Chr5), which was primarily trisomic and exhibited a high frequency of segmental aneuploidy comprising the two left arms of Chr5 flanked by a single centromere, an isochromosome 5L [i(5L)]. Sometimes, i(5L) was attached to a homolog of Chr5. The left arm of Chr5 houses *ERG11* and the *TAC1* gene, which encodes a transcription regulator of drug efflux pumps. Amplification of these two genes was determined to be the major mechanism responsible for increased drug resistance in i(5L) aneuploids. The levels of azole resistance were independently and additively mediated by the copy number of *ERG11* and *TAC1* genes [24]. The acquisition of i(5L) accompanied by FLC resistance also occurred commonly in clinical settings during FLC treatment [24], and such genomic aneuploidy was reproducible in the laboratory following short exposure to FLC. A correlation between formation of the i(5L) and azole resistance is evident, since it is only observed in the azole-treated population and not in the untreated population of the same strain at any given time point [4]. Like in the *C. glabrata* case of FLC-induced aneuploidy, i(5L) formed under FLC stress was lost during proliferation in an environment free of FLC. These findings underscore the genomic fluidity of *C. albicans* and confirm how drug-induced aneuploidy can provide a fitness advantage [4], [20].

## How Does FLC-Induced Aneuploidy Contribute to the Emergence of Transient Azole Resistance in *Cryptococcus*?

An adaptive mechanism of drug resistance against azoles in *C. neoformans* called “heteroresistance,” later found to be due to azole-associated acquisition of aneuploidy, was first reported in clinical isolates from two patients: one undergoing azole maintenance therapy and the other with no exposure to antifungal drugs [25]. Screening of over 100 *C. neoformans* and more than 40 *C. gattii* strains isolated before the advent of azoles confirmed both species to be innately heteroresistant to FLC [9], [26], and this resistance was lost upon release from drug stress [5], [27]. The resistant subpopulations that emerged at drug levels higher than the strain's MIC for FLC almost always were disomic (chromosome present in two copies) for Chr1, and further elevations of the drug level additionally resulted in Chr4 disomy [5], [27]. *C. neoformans* is a haploid yeast with 14 chromosomes, and the clones that emerged at the highest drug level tested were found to contain disomies of as many as four chromosomes, Chr1, 4, 10, and 14. Chr1 contains *ERG11* and also *AFR1*, which encodes an ATP-transporter efflux pump associated with azole resistance. Translocation of *ERG11* to another chromosome or deletion of *AFR1* from Chr1 provided evidence that amplification of these two genes due to disomy of Chr1 was the major mechanism responsible for resistance under FLC stress. Whether FLC induces disomies of specific chromosomes in *C. neoformans* or creates a selective environment in which aneuploid clones survive has not been clearly addressed. It is feasible that aneuploid clones were derived from the minor population of transiently formed diploid cells. A small fraction of the cells exposed to FLC levels above their MIC for 4 hr contained multiple nuclei (unpublished observation), and some of these cells may transiently have become polyploids. Adaptive polyploidy has been reported in mammalian systems when human HL-60 cells were treated with SKF 10496, which targets the *ERG11* homolog in cholesterol biosynthesis, thus depriving the cells of cholesterol which is essential for mitosis [28]. It is possible that the fraction of azole-induced polyploids undergo a rapid loss of extra chromosomes, while those that retain aneuploidy of chromosomes carrying genes relevant for azole resistance are able to selectively survive under FLC stress.

**Figure 1:**
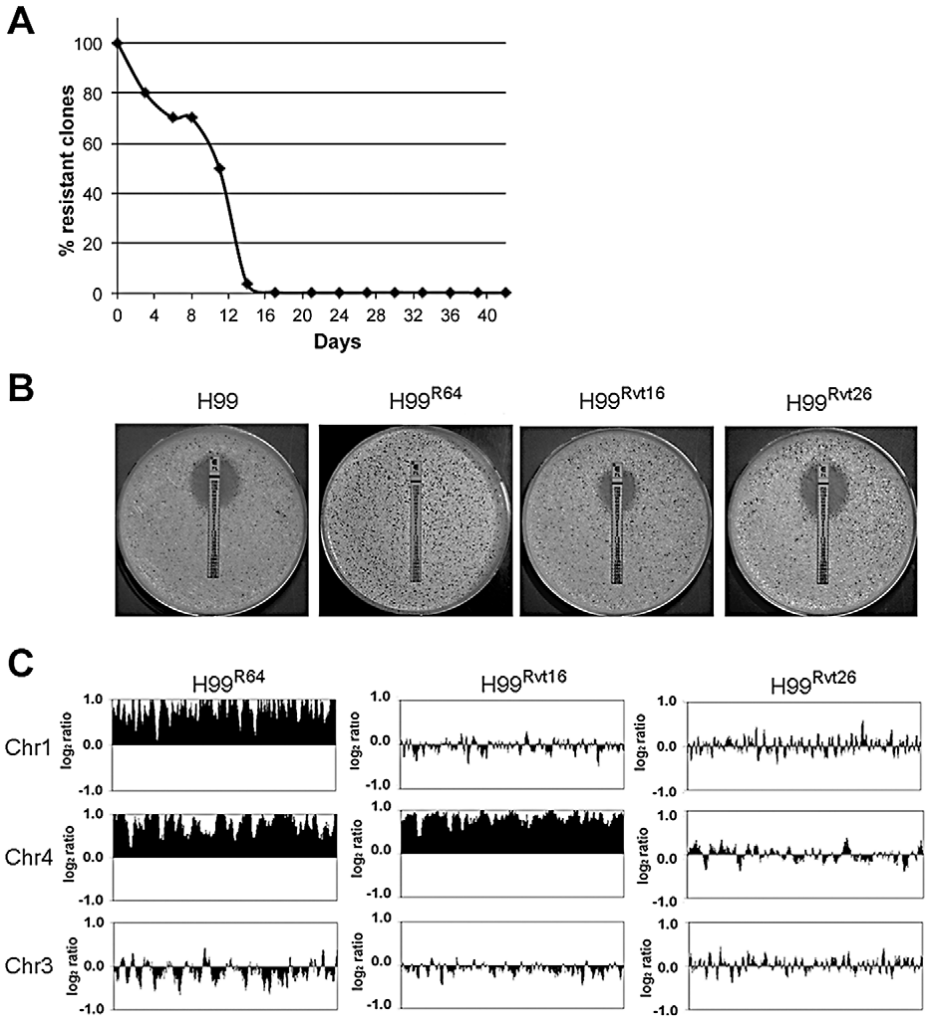
Aneuploidy formation and FLC resistance in the *C. neoformans* strain H99. (A) The percentage of FLC-resistant population in H99^R64^ (resistant at 64 ug/ml FLC) decreases during daily transfer in drug-free media. (Adapted from Sionov et al. [9].) (B) E-test showing FLC-resistance phenotype of H99 wild type, H99^R64^, H99^Rvt16^, and H99^Rvt26^. Complete reversion of drug sensitivity to wild-type levels occurs by day 26. (C) CGH plots showed duplication of Chr1and 4 in resistant clones proliferating at 64 ug/ml FLC (H99^R64^), loss of Chr1 disomy by day 16 (H99R^vt16^) for subcultures grown in drug-free media, and complete loss of disomic chromosomes by day 26 (H99^Rvt26^). (Adapted from Sionov et al. [5].)

Unlike in *C. albicans*, duplications of Chr1 in *C. neoformans* encompass the whole chromosome without forming segmental isochromosomes. Since *ERG11* and *AFR1* are on opposite arms of Chr1 in *C. neoformans*, duplication of Chr1 in its entirety could have offered a selective advantage during evolution of a resistant population under FLC stress. It may also be due to the *C. neoformans* genome being less plastic than *C. albicans*. Upon release from drug stress, the acquired extra copies of chromosomes are lost sequentially in *C. neoformans*: Chr1 disomy is lost first, followed by the loss of the disomy of the considerably smaller Chr4 (Figure 1). Losing the largest extra chromosomes more readily than smaller ones is consistent with the finding that eukaryotic systems better tolerate aneuploidy of smaller chromosomes [1]. The loss of aneuploidy in *C. neoformans* appears to be associated with the apoptosis-inducing factor Aif1, since inactivation of the *AIF1* gene rendered the Chr1 disomy to be stable in the absence of the drug [29]. What makes Chr4 the second most common chromosome to be disomic under FLC stress? Analysis and characterization of genes on Chr4 revealed that maintenance of endoplasmic reticulum (ER) integrity is the major reason for Chr4 duplication under FLC stress [27], [30].

## How is ER Important in the Emergence of Aneuploidy in *Cryptococcus*?

**Figure 2:**
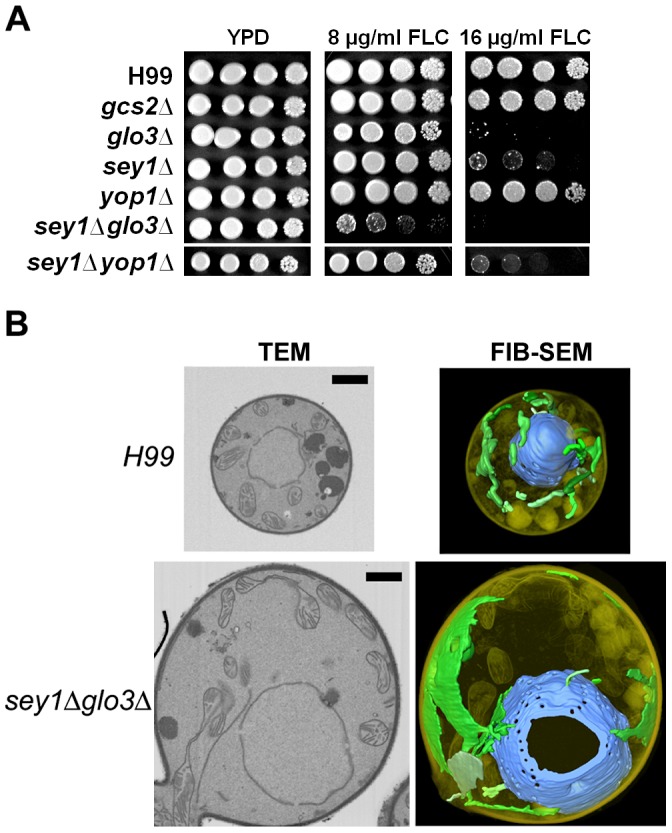
Importance of ER integrity and resistance to FLC. (A) Spot assay showing FLC sensitivity of the strains with deletion of ER integrity-associated genes. (Adapted from Ngamskulrungroj et al. [27], [30].) (B) Focused ion beam-scanning electron microscopy (FIB-SEM) shows severe perturbation of ER network in a *sey1Δglo3Δ* double deletant compared to H99. ER and nuclei were pseudocolored green and blue, respectively (right column). Left column shows orthoslices representing central sections by transmission electron microscopy. The cells of *sey1Δglo3Δ* are considerably larger than the wild type or deletant of a single gene. Bar = 1 µm. (Adapted from Ngamskulrungroj et al [27].)

Aneuploidy of Chr4, in addition to Chr1, was found in a majority of the *C. neoformans* clones that could resist very high FLC concentrations (≥64 µg/ml). Chr4 lacks ergosterol biosynthesis-related genes or homologs of efflux pumps that affect Chr4 disomy formation [27]. Since azoles perturb the cell membrane integrity, Ngamskulrungroj et al. focused on six Chr4 genes that are associated with membrane composition/integrity and analyzed their role in FLC resistance and formation of Chr4 disomy [27]. Of the six, three genes that are involved in the maintenance of ER integrity, *SEY1*, *GLO3* and *GCS2*, were found to be important for aneuploidy formation under FLC stress. In addition, deletion of *SEY1* and *GLO3* resulted in significantly higher susceptibility to FLC (Figure 2A), and deletants showed various degrees of ER perturbation accompanied by 40% to 90% reductions in the frequency of FLC-associated formation of disomy. Importance of ER integrity in FLC-associated drug resistance via disomy formation was further confirmed by an increase in Chr3 disomy when *SEY1* or *GLO3* was translocated to Chr3. Moreover, *SEY1* and *GLO3* double deletions caused severe perturbation of the ER network (Figure 2B) and abolished disomy for both Chr1 and Chr4 under FLC stress. Those double deletants were extremely sensitive to FLC, and the clones resistant to levels of FLC higher than their MIC revealed a monosomic Chr1 with segmental amplification only in the small region surrounding the *ERG11* gene [27]. Similar results were obtained when *SEY1* and the Chr7-inhabiting *YOP1* gene, which encodes a Sey1-interacting ER curvature maintenance protein, were both deleted [30]. Since FLC disrupts the biosynthesis of ergosterol, which is produced in the ER and delivered to plasma membrane [31], [32], it is likely that increases in dosage of the genes relevant for ER integrity provide increased fitness under FLC stress. The mechanism of how ER influences aneuploidy formation under azole stress remains unknown.

## Concluding Remarks

Aneuploidy, which provides increased fitness in *Candida* and *Cryptococcus* under azole stress, has received considerable attention, but the mechanism of chromosome missegregation that causes azole-associated aneuploidy has not been characterized in either fungus. However, the mechanism of adaptive azole resistance in the two species appears to overlap: amplification of the chromosome bearing *ERG11*, the target of azoles, and either the efflux pumps or their regulator accounts for the drug resistance. In *C. neoformans*, duplication of the chromosome that houses factors responsible for the maintenance of ER integrity also provides a fitness advantage under azole stress. Since sterol is essential for the proliferation of fungal cells and is synthesized mainly in the ER before being delivered to the plasma membrane, it is understandable how *C. neoformans* benefits through amplification of ER-associated genes under azole stress. Further investigation is warranted to see if the relationship between ER integrity and the azole-associated emergence of aneuploidy is common among fungi.
